# Spectroscopic characterization of electronic structures of ultra-thin single crystal La_0.7_Sr_0.3_MnO_3_

**DOI:** 10.1038/s41598-021-84598-8

**Published:** 2021-03-04

**Authors:** Chun-Chien Chiu, Yao-Wen Chang, Yu-Cheng Shao, Yu-Chen Liu, Jenn-Min Lee, Shih-Wen Huang, Wanli Yang, Jinghua Guo, Frank M. F. de Groot, Jan-Chi Yang, Yi-De Chuang

**Affiliations:** 1 Department of Physics, National Cheng Kung University, Tainan, 701 Taiwan; 2 Advanced Light Source, Lawrence Berkeley National Laboratory, Berkeley, CA 94720 USA; 3 MAX IV Laboratory, Lund University, P. O. Box 118, 221 00 Lund, Sweden; 4 Debye Institute for Nanomaterials Science, Utrecht University, Universiteitsweg 99, 3584 CG Utrecht, The Netherlands

**Keywords:** Physics, Materials science, Condensed-matter physics

## Abstract

We have successfully fabricated high quality single crystalline La_0.7_Sr_0.3_MnO_3_ (LSMO) film in the freestanding form that can be transferred onto silicon wafer and copper mesh support. Using soft x-ray absorption (XAS) and resonant inelastic x-ray scattering (RIXS) spectroscopy in transmission and reflection geometries, we demonstrate that the x-ray emission from Mn 3*s*-2*p* core-to-core transition (3*s*PFY) seen in the RIXS maps can represent the bulk-like absorption signal with minimal self-absorption effect around the Mn *L*_3_-edge. Similar measurements were also performed on a reference LSMO film grown on the SrTiO_3_ substrate and the agreement between measurements substantiates the claim that the bulk electronic structures can be preserved even after the freestanding treatment process. The 3*s*PFY spectrum obtained from analyzing the RIXS maps offers a powerful way to probe the bulk electronic structures in thin films and heterostructures when recording the XAS spectra in the transmission mode is not available.

## Main

Rare-earth/alkaline-earth manganese oxides R_1−x_A_x_MnO_3_ (R = La, Ce …; A = Ca, Sr, Ba) have been extensively studied over the past decades due to their rich phase diagrams that exhibit different magnetic and transport ground states and electronic ordering phenomena that are manifested by the interactions like electron itinerancy, Coulomb repulsion, Hund’s rule coupling, double-exchange, super-exchange, etc. The proximity of the energy scales of these interactions and their couplings to lattice, charge, spin, and orbital degrees of freedom permit the tuning of disparate ground states through a variety of perturbations to induce colossal responses, thereby offering great potentials in multifunctional applications^1–4^. Of particular interest in these manganites is the wide-bandwidth La_1-x_Sr_x_MnO_3_ (LSMO) with x = 0.3, whose fully spin-polarized metallic ground state at ambient condition (Curie temperature Tc ~ 360 K) has been proposed as the candidate for the spintronic device applications^5,6^.

However, to facilitate the incorporation of these manganites into actual devices, their form factors need to be flexible and integrable into different platforms. Although manganites can be routinely grown on top of oxide substrates using techniques such as pulsed laser deposition (PLD) and molecular beam epitaxy (MBE) to attain the precision of layer-by-layer growth, these hard substrates nevertheless present challenges to certain applications that require flexibility and bendability, notably in the wearable electronics. Recently, it has been shown that some transition metal oxides (TMOs) can be fabricated on flexible supports like mica^7,8^, polymers^9^, or even in the freestanding form to overcome this substrate constraint^10–13^, and it will be of paramount importance to verify that these TMOs retain their bulk electronic properties after the meticulous growth and preparation processes. In this letter, we report the soft x-ray spectroscopic studies on crystalline La_0.7_Sr_0.3_MnO_3_ in freestanding form (LSMO-FS) and on the SrTiO_3_ (STO) substrate (La_0.7_Sr_0.3_MnO_3_/SrTiO_3_ or LSMO/STO, the reference sample). We show that these manganites have similar bulk electronic structures and demonstrate that the Mn 3*s*-2*p* core-to-core transition (3*s*PFY) besides the proposed inverse partial fluorescence yield (*i*PFY) from oxygen emission can be used to reliably obtain such information.

**Figure 1:**
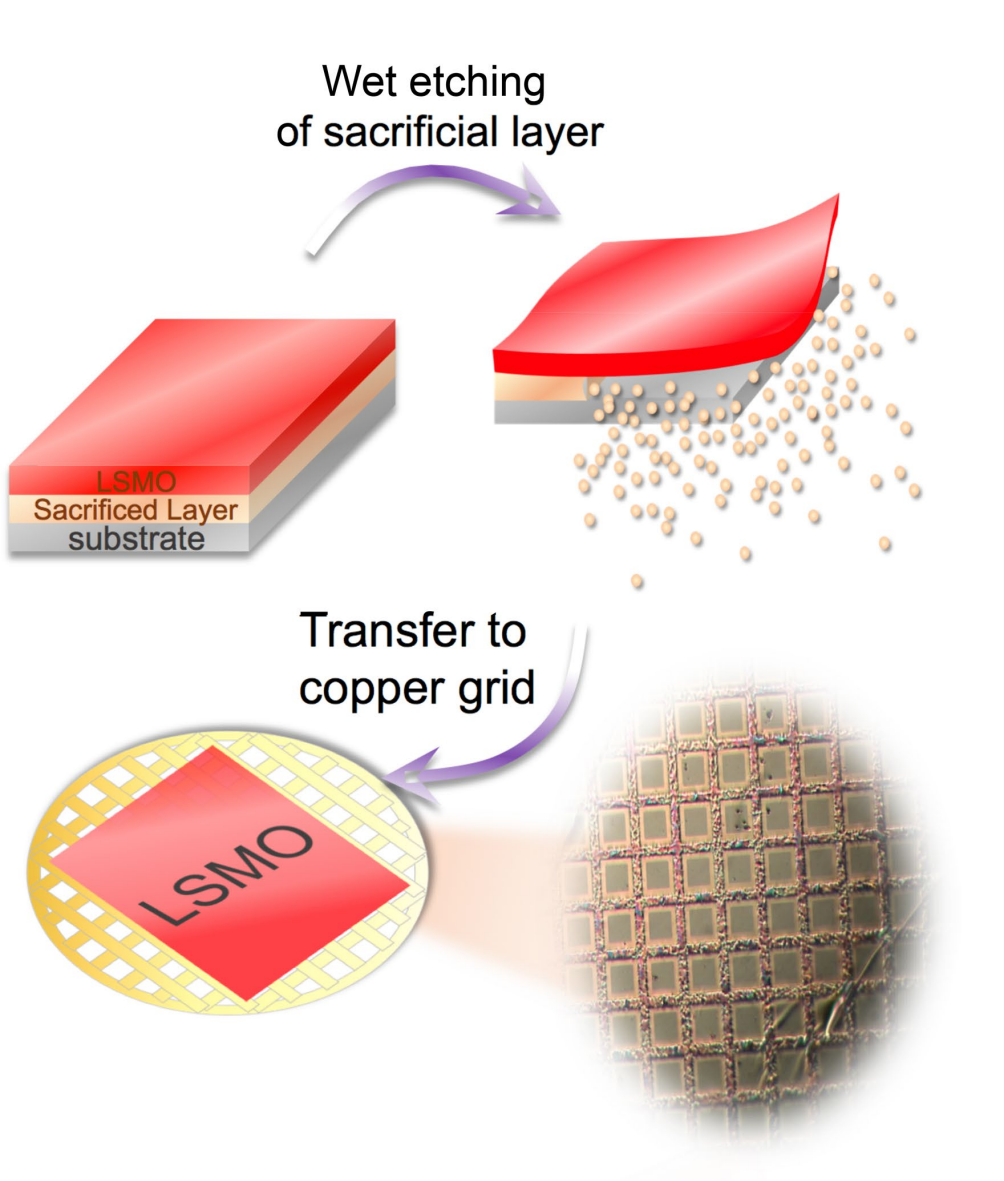
Schematic illustration of the fabrication of freestanding LSMO thin film. The bottom-right panel shows the microscope image of a freestanding film that is transferred onto a 400 mesh Cu grid.

The freestanding LSMO thin film (LSMO-FS) was prepared by the method illustrated in Fig. 1. First, the high-quality La_0.7_Sr_0.3_MnO_3_ (100 nm) and sacrificial YBa_2_Cu_3_O_7_ (25 nm) layers were epitaxially deposited on the (001)-oriented SrTiO_3_ substrate by PLD to form the LSMO/YBCO/STO structure. After coating the top LSMO layer with the protective Poly (methyl methacrylate) (PMMA), the assembly was immersed in light hydrochloric acid to etch away the YBCO layer, releasing the PMMA/LSMO into the solution that can be transferred onto rigid supports for further process to remove the PMMA coating. The reference LSMO/STO sample was fabricated following the same PLD process. More details about the fabrication and characterizations of the as-grown and similar LSMO-FS films can be found elsewhere^13^.

**Figure 2:**
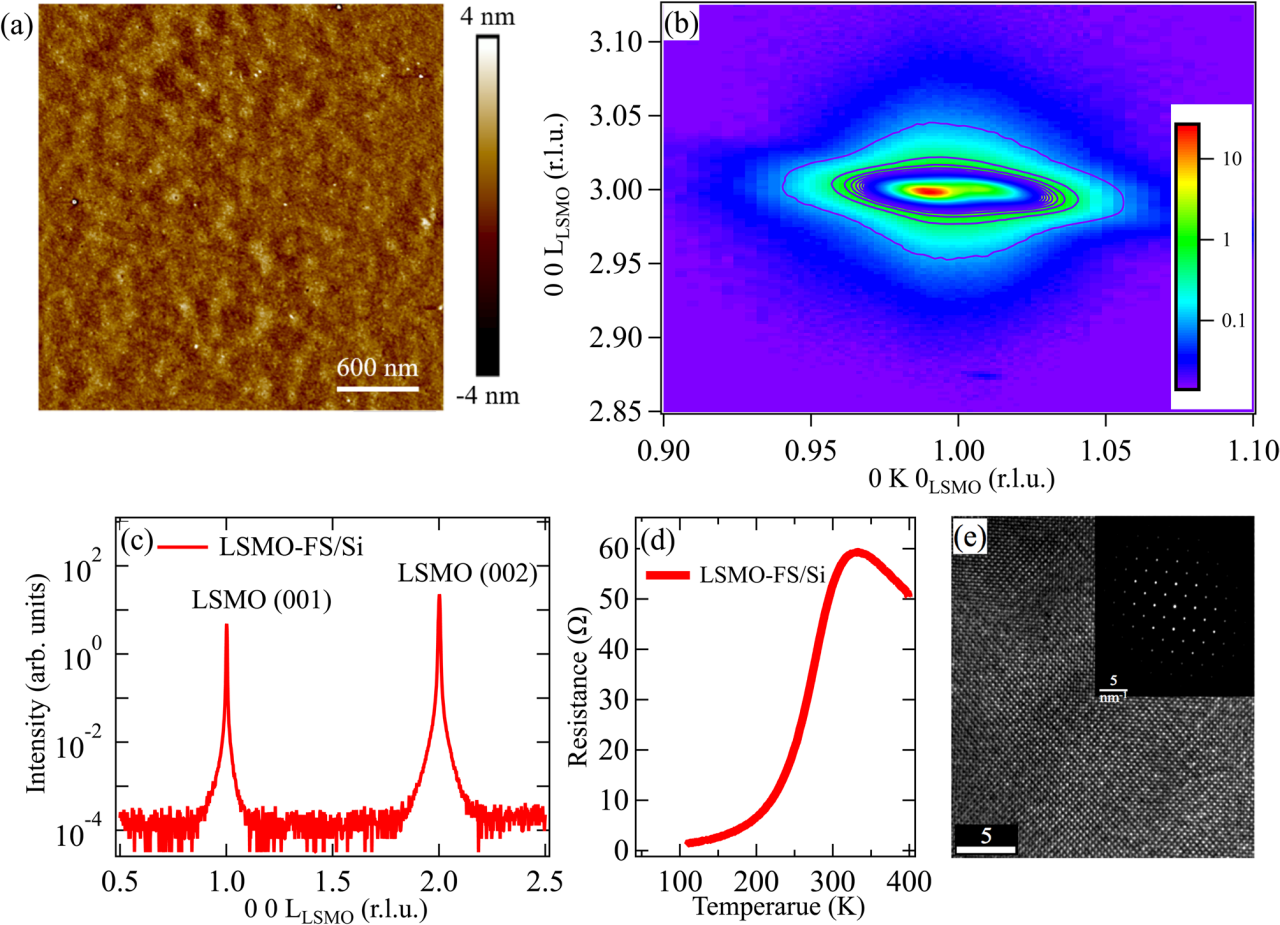
Characterization of the freestanding LSMO thin film on Si substrate (LSMO-FS/Si). (**a**) Surface morphology from AFM. (**b**) The reciprocal space mapping around (013) diffraction peak. (**c**) XRD θ-2θ scan along the (00L) direction. (**d**) The resistance curve obtained by four-point method. (**e**) TEM image. The inset shows the electron diffraction.

We carried out a series of characterizations on the freestanding LSMO on the Si wafer support to validate the film quality before performing the x-ray measurements on those that are supported by Cu mesh (to permit transmission geometry measurements). The atomic force microscope (AFM) was used to examine the morphology of LSMO-FS film. The AFM image in Fig. 2a shows a smooth topography without any observable pits or laser ablated particles, suggesting no structural degradation after the freestanding treatment process. The reciprocal space mapping (RSM, Fig. 2b) around the (013) diffraction peak confirms the single phase without impurities, and the estimated in-plane *b*-axis lattice constant is ~ 3.908 ± 0.018 Å (pseudo cubic notation). The x-ray diffraction (XRD) θ-2θ scan along the (00L) direction in Fig. 2c displays a clear background, indicating that neither substrate effect nor impurity phases is present in the freestanding thin film. The out-of-plane *c*-axis lattice constant estimated from the XRD curve is 3.887 ± 0.016 Å (pseudo cubic notation), in agreement with the bulk parameters^14^. The temperature-dependent resistance was measured using the four-terminal method, see Fig. 2d. The resistance curve exhibits the classical metal–insulator phase transition around 355 K, comparable to that of the single crystal (T_C_ = 369 K)^15^. Figure 2e shows the plane view transmission electron microscopy (TEM) image along the [001] zone axis. Absence of observable defects in this TEM image also corroborates the findings from AFM.

Although the transport properties and structural parameters of LSMO-FS film are consistent with the reference LSMO/STO film^13^, it is important to verify that they have similar electronic structures; or equivalently, the electronic structures remain minimally perturbed after the freestanding treatment process. To address this issue, we resort to the soft x-ray absorption (XAS) and resonant inelastic x-ray scattering (RIXS) spectroscopy at Mn *L*_2,3_-edge, which correspond to the on-site transitions from Mn 2*p*^6^3*d*^n^→2*p*^5^3*d*^n+1^. The measurements on LSMO-FS and LSMO/STO were carried out at BL 8.0.1 at the Advanced Light Source (ALS), Lawrence Berkeley National Laboratory, using the qRIXS endstation^16^. A high throughput modular x-ray spectrometer placed at 140° back-scattering angle was used to record the RIXS spectra when the excitation photon energy was scanned across the Mn *L*_2,3_-edge (the compilation of these RIXS spectra is termed the RIXS map)^17^. During the measurements, the LSMO-FS and LSMO/STO films were placed in the normal incidence geometry with incident x-ray beam less than 10° from the sample surface normal. There was no specific orientation alignment for LSMO-FS; but for the reference LSMO/STO, the Mn–O bond direction was in the horizontal scattering plane. The photon polarization was kept in the horizontal scattering plane (π-polarization) and the beamline energy resolution was set to 0.3 eV. The combined energy resolution (beamline plus spectrometer) for RIXS measurements determined from the full width at half maximum (FWHM) of the elastic peak was 0.4 eV. The sample-to-ground drain current was used for the total electron yield (TEY) mode measurement, whereas a GaAsP photodiode was used to record the total fluorescence yield signal (TFY) at two angles: 0° for the transmission geometry (trans, for LSMO-FS only) and 150° back-scattering angle for the reflection geometry (ref, for both samples). For normalization, the XAS spectra were first subtracted by a linear background from fitting their respective pre-edge region below the Mn *L*_3_ edge, and then the edge jump that is 15 eV above the Mn *L*_2_ edge was normalized to 1. To calculate the branching ratio, BR = I(*L*_3_)/(I(*L*_2_) + I(*L*_3_)) where I(*L*_3_) and I(*L*_2_) are the integrated spectral area over the Mn *L*_3_ and *L*_2_ edges, respectively, the edge jump was further removed from the XAS spectra by subtracting an arctangent function^18^. For the nomenclature of XAS spectra, they will be called in the following fashion: (geometry)-(mode)-XAS; for example, trans-XAS means the XAS spectrum recorded in the transmission geometry. All data shown here were recorded at 300 K.

**Figure 3:**
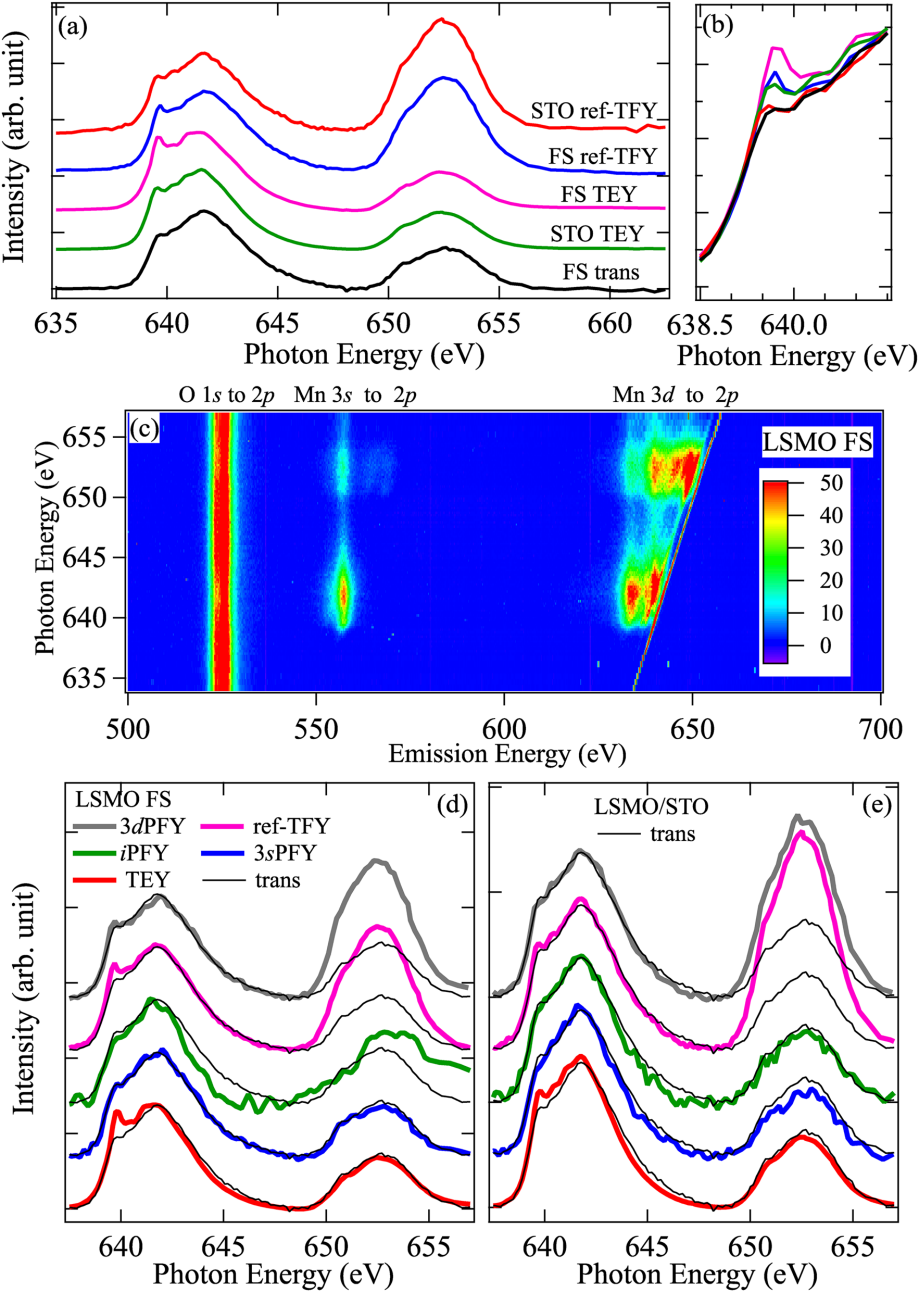
(**a**) Mn *L*_2,3_-edge x-ray absorption spectra (XAS) of LSMO thin film in freestanding form (FS) and on the STO substrate (STO) measured in TEY and TFY modes. For the FS sample, the measurements were carried out in both reflection (ref) and transmission (trans) geometry. (**b**) A magnified view of low energy shoulder around the *L*_3_-edge. (**c**) 2D RIXS map of LSMO-FS with incident photon energy scanned across Mn *L*_*2,3*_-edge. Features associated with different emission channels are labeled on top of the figure. (**d**,**e**) Comparison of TEY (red), TFY (in reflection geometry, pink), partial (3*s*PFY, blue; 3*d*PFY, gray)/inverse-partial yield spectra (*i*PFY, green) from the RIXS maps, and the transmission XAS spectrum (thin black) from LSMO-FS for (**d**) LSMO-FS and (**e**) LSMO/STO samples.

Figure 3a shows the XAS spectra of LSMO-FS and LSMO/STO films recorded in TEY and TFY modes in the reflection and transmission geometries. For comparison, all spectra are further normalized to the maximum of *L*_3_ edge at 642 eV. From this figure, one can see that the TEY-XAS spectra of both films are very similar except around the pre-edge region (639.6 eV) where LSMO-FS exhibits a more pronounced spectral feature (see the magnified view in Fig. 3b). The ref-TFY-XAS spectra of these samples are also very similar, although the spectral weight at the *L*_2_ edge displays some discrepancy. Since the TFY channel has a much larger probing depth (on the order of 100 nm) than the TEY channel (on the order of few nm)^19^, the similarity between the ref-TFY-XAS spectra implies very similar bulk electronic structures between these films. Although the ref-TFY-XAS spectrum is more bulk sensitive, it suffers from the strong self-absorption effect at Mn *L*_3_ edge that renders the branching ratio BR much smaller than the TEY-XAS spectrum^20^. This distorted BR can be problematic if used to estimate the strength of local spin–orbit coupling (SOC)^18,21^. The self-absorption effect also prohibits us from claiming that these films have similar bulk electronic structures. On the other hand, the enhanced pre-edge feature in the TEY-XAS spectra is known to originate from the Mn^3+^ state^22,23^, and the surface sensitivity of TEY-XAS spectra indicates more excess oxygen vacancies at the surface of LSMO-FS film, presumably caused by the wet chemical etching process.

The LSMO-FS film allows us to perform the XAS measurement in the transmission mode (trans-XAS), which by definition is the true bulk absorption. Interestingly, if comparing the trans-XAS spectrum with the TEY-XAS and ref-TFY-XAS spectra of LSMO-FS, one can see that the surface-related Mn^3+^ feature is much reduced in the trans-XAS spectrum. In addition, its BR is almost the same as the TEY-XAS spectrum. Such observation reflects the general consensus that both TEY-XAS and ref-TFY-XAS spectra only partially represent the true bulk absorption signal. It has been suggested that besides the aforementioned TEY and TFY XAS, other x-ray and photoelectron emission channels can provide complimentary information for the bulk electronic structures^20,24–27^. With the high throughput soft x-ray spectrometer, we are able to record the RIXS spectra with the excitation photon energy spanning across the Mn *L*_2,3_-edge in a reasonable time frame (9 min per RIXS spectrum and 0.15 eV incident photon energy step). The resulting RIXS map for LSMO-FS film is shown in Fig. 3c.

In this RIXS map, three emission features can be clearly identified. From left (lower emission energy) to right (higher emission energy), they correspond to the following transitions: O 2*p*→1*s* (515 eV ~ 535 eV, used to derive the inverse partial fluorescence yield spectrum or *i*PFY by inverting the spectral profile), Mn 3*s*→2*p* (550 eV ~ 575 eV, for core-core partial fluorescence yield spectrum or 3*s*PFY) and Mn 3*d*→2*p* (620 eV ~ 665 eV, 3*d* partial fluorescence spectrum or 3*d*PFY)), respectively. By integrating the emission intensity within individual energy windows, we can obtain different partial fluorescence yield (PFY) spectra as shown in Fig. 3d^28^. In this figure, we also overlay the TEY-XAS (red), trans-XAS (thin black), and ref-TFY-XAS (pink) spectra of LSMO-FS film for comparison. We see that all PFY and TFY spectra show a much weaker pre-edge feature around 639.6 eV, consistent with their bulk sensitivity with photon emission. Although these spectra exhibit subtle variation around the pre-edge region due to their varying degree of probing depth and self-absorption effect^29,30^ the biggest contrast is in their BR value (see summary in Table 1). The BR for 3*d*PFY is ~ 0.49, only slightly smaller than the ref-TFY-XAS and *i*PFY; however, this value is much smaller than the TEY and 3*s*PFY and the nominal value around 2/3 (~ 0.68 if considering the sample doing level), see Table 1. Therefore, 3*d*PFY still experiences a strong self-absorption effect like TFY. *i*PFY was previously proposed to best represent the bulk absorption; however, for the case of LSMO-FS, its BR value still deviates from the nominal value. Furthermore, the *i*PFY spectrum also displays poor statistics likely due to the lower cross-section and excessive oxygen vacancies in this sample. Comparison in Fig. 3d shows that 3*s*PFY bears the closest resemblance to the trans-XAS spectrum with the nearly identical BR, implying that it is the most direct probe for the bulk electronic structures besides trans-XAS spectrum. In addition, we also note that 3*s*PFY channel has a higher yield than the *i*PFY^26^ such that it has better statistics for very thin films.

**Table 1:** The probing depth of different detection modes.

Detection mode	Depth (nm)	Note	Branching ratio LSMO-FS; LSMO/STO
Transmission (Trans)	~ 100		0.713
TEY	~ 5	Surface	0.71/0.71
*i*PFY	~ 50	No saturation, low signal	0.543/0.697
3*s*PFY	~ 50	Saturation	0.719/0.733
3*d*PFY	~ 50	Saturation, state-dependent decay	0.468/0.496
TFY	~ 50	Mainly 3*d*PFY	0.509/0.455

With this finding, we now look at the PFY spectra produced from the LSMO/STO RIXS map (data not shown) in Fig. 3e. For comparison, we also overlay these spectra with the trans-XAS from LSMO-FS. In this figure, we also see that 3*s*PFY spectrum gives the best agreement with the trans-XAS spectrum. Interestingly, the 3*s*PFY of LSMO/STO has an even smaller pre-edge spectral weight, which is consistent with the findings from the TEY-XAS spectra in Fig. 3b that points to less surface oxygen vacancies in LSMO/STO. The agreement between the trans-XAS spectrum from LSMO-FS and the 3*s*PFY spectrum from both LSMO-FS and LSMO/STO substantiates the claim that the LSMO-FS film retains its bulk electronic structures even after the freestanding treatment process, although its surface may possess additional oxygen vacancies that warrants further fine-tuning in the treatment to reduce them.

The potential of using 3*s*PFY technique to study the electronic structures has been explored theoretically^25^ and experimentally^26^. Miedema et al*.*^25^ pointed out that although 3*s*PFY spectrum can be representative to the true XAS spectrum, certain care must be paid to the experimental configuration. For example, with the capability to analyze the photon polarizations on both incident (polarization control on the X-ray source) and emission side (with polarimeter on the spectrometer) and a proper placement of spectrometer to enhance the contrast, one may detect the linear dichroism between the parallel and crossed polarization channels. This can be advantageous for studying the respective orbital contributions in the resulting 3*s*PFY spectrum; however, it can lead to certain error in estimating quantities such as BR. In that regard, one needs to perform the polarization averaging to correct this error. In practice, this can be accomplished by averaging the incident (measurements with both linear vertical and horizontal polarizations) and emission (not using the polarimeter) polarizations and varying the sample orientations if needed (for single crystalline sample without O_h_ symmetry). Experimentally, Busse et al.^26^ showed that 3*s*PFY yields a closer BR value to the nominal value compared with other techniques such as 3*d*PFY, *i*PFY, and TFY in the reflection geometry; however, the 3*s*PFY BR value still deviates from the nominal one. This is also seen in the current study, and the discrepancy is likely due to several factors such as saturation (much weaker in the 3*s*PFY channel), the fixed measurement geometry, the treatment of spectral background, etc. Nevertheless, these factors do not impact the main conclusion that 3*s*PFY is a suitable bulk probe for electronic structures than ref-TFY-XAS or 3*d*TFY.

Besides using the RIXS maps to produce the 3*s*PFY spectra to obtain the bulk absorption information, these maps also reveal contrasting Coster-Kronig (C-K) transitions between 3*s*-2*p* and 3*d*-2*p* channels^31^. The C-K transitions at 3*d*-2*p* channel have been examined in the Co^2+^ systems^32^, but to our knowledge, this is the first report at 3*s*-2*p* channel in solid state TMO. Notably, besides the contrasting self-absorption effect that complicates the measurements^33^, the multiplets in the 3*d*-2*p* transition are dominating over the C-K related features while the situation is reversed in the 3*s*-2*p* transition. Such distinct behavior is related to different final state configurations when the photon energy is tuned to the *L*_2_ edge to activate the C-K transition. Taking the RIXS map in Fig. 3c as an example, around the 3*d*-2*p* channel (~ 650 eV emission energy), the diagonal and vertical features correspond to (neglecting the charge-transfer) the transitions 3*d*^4^→3*d*^5^*L*_2_→3*d*^*4*^ and 3*d*^4 ^→3*d*^5^*L*_2_→(C-K) 3*d*^*5*^*L*_*3*_→3*d*^4^, respectively (*L* denotes the core–hole). On the other hand, around the 3*s*-2*p* channel (~ 570 eV emission energy), they correspond to 3*d*^4^→3*d*^5^*L*_2_→ *3s*^*1*^*3d*^*5*^ and 3*d*^4^→3*d*^5^*L*_2_→(C-K) *3d*^*5*^*L*_3_→x*3s*^*1*^*3d*^*5*^, respectively. Because of this difference, the integrated spectral weight over respective emission energy window can be used to gauge the degree of covalency and study the decay rates of different channels, such as 2*p*-3*d*, *L*_3_-*L*_2_ Auger, and 2*p*-3*s* transitions. In addition, the final state configuration of 3*s*^1^3*d*^5^ can be used to explore the internal *s*-*d* interaction. Further studies on manganites will be reported elsewhere.

In summary, we have successfully prepared the freestanding La_0.7_Sr_0.3_MnO_3_ thin film and carried out soft x-ray XAS and RIXS measurements on this LSMO-FS film and a reference LSMO/STO film. We show that the bulk electronic structures of LSMO-FS can be well preserved after the wet chemical etching process to produce the freestanding film despite having more surface oxygen vacancies. Although a similar use of 3*s*PFY spectrum derived from RIXS maps to study the bulk electronic structures can be found in *operando* chemistry with using primarily a jet system to deliver the dilute targets, our work in LSMO single crystal constitutes an extensive evaluation of the pros and cons of different TFY, PFY, and TEY mode of XAS in correlated oxides. The use of 3*s*PFY mode to obtain the bulk-like XAS spectrum is of particular attractive for certain class of materials. For example, heterostructural oxides grown on (or transferred to) flexible support can be used for next generation electronic devices, and understanding the electronic structures of buried layers can be the target application for this technique^8^. Note that due to the minimal self-absorption in 3*s*PFY mode relative to ref-TFY-XAS and 3*d*PFY, one expects that this technique will be particular useful for elements like Cr, Mn, Fe in oxides whose *L* absorption edge is close to O *K*-edge. Besides the practical aspect of this technique, we show that the improved energy resolution in RIXS spectroscopy in this work allows us to examine the spectral features associated with the Coster-Kronig transition and offers an attractive approach to determine its strength over a wide class of materials that are inaccessible to photoelectron spectroscopy.
